# Insights into permanent pacemaker implantation following TAVR in a real-world cohort

**DOI:** 10.1371/journal.pone.0204503

**Published:** 2018-10-17

**Authors:** Tobias Tichelbäcker, Leonard Bergau, Miriam Puls, Tim Friede, Tobias Mütze, Lars Siegfried Maier, Norbert Frey, Gerd Hasenfuß, Markus Zabel, Claudius Jacobshagen, Samuel Sossalla

**Affiliations:** 1 Clinic for Cardiology & Pneumology, University Medical Center Goettingen, Goettingen, Germany; 2 DZHK (German Center for Cardiovascular Research), partner site Goettingen, Goettingen, Germany; 3 Heart Center Cologne, University Hospital Cologne, Cologne, Germany; 4 Department of Medical Statistics, University Medical Center Goettingen, Goettingen, Germany; 5 Department for Internal Medicine II, Cardiology, Pneumology, Intensive Care, University Hospital Regensburg; Regensburg, Germany; 6 Department for Internal Medicine III, University Hospital Kiel, Kiel, Germany; Klinikum Region Hannover GmbH, GERMANY

## Abstract

**Background:**

Permanent pacemaker implantation (PPI) following TAVR is a frequent post interventional complication and its management remains controversial.

**Objective:**

We sought to elucidate the electrophysiological, procedural, and clinical baseline parameters that are associated with and perhaps predict the need for PPI after TAVR in a heterogeneous-valve-type real-world cohort.

**Methods:**

Overall, 494 patients receiving TAVR at our center from April 2009 to August 2015 were screened. ECG analyses and clinical parameters were collected prospectively.

**Results:**

Overall, 401 patients in this all-comers real-world TAVR cohort with a PPI rate of 16% were included. The mean age was 82 years, and the mean duration to PPI was 5.5 days. A large proportion of Edwards SAPIEN valves (81%), DirectFlow, CoreValve, and Portico were implanted. The main indications for PPI were atrioventricular (AV) block III, AV-block Mobitz type II, bradycardic atrial fibrillation and persistent sinus bradycardia. Between groups with and without PPI, significant differences were noted in the prevalence of post TAVR balloon dilatation, resting heart rate, QRS interval, PR interval with a cut-off of >178 ms, left anterior fascicular block and RBBB in univariate analyses. In the subsequent multiple regression analysis, post TAVR balloon dilatation and a PR interval with a cut-off of >178 ms were significant predictors of PPI.

**Conclusion:**

This real-world cohort differs from others in its size and heterogeneous valve selection, and indicates for the first time that patients with post balloon dilatation or prolonged PR interval are at a higher risk for pacemaker dependency after TAVR.

## 1 Introduction

Aortic valve stenosis is the most common valvular heart disease in industrialized nations[1]. Transcatheter aortic valve replacement (TAVR) has become a therapeutic option for patients with severe symptomatic aortic stenosis at high surgical risk[2]. In Germany alone, nearly 16,000 patients were treated with TAVR[3] between 2013 and 2015, and implantation rates are increasing worldwide. Excellent results obtained in clinical trials have initiated a reassessment of the recommendations for the treatment of aortic stenosis and hence may trigger a wider use of TAVR.

High degree AV-block requiring permanent pacemaker implantation (PPI) and paravalvular leakage account for the most common complications following TAVR. The PPI rates have varied from 2–51% in the current literature[4][5][6]. New valve designs have been developed and are now commonly used in order to minimize the risk of paravalvular leakage, which constitutes an important predictor of poor outcomes after TAVR[7][8]. Early results of the third-generation devices showed an increase in conduction disturbances (e.g., Edwards SAPIEN 3 (ES3), (Edwards Lifesciences, Inc., Irvine, CA, USA) requiring PPI[9], probably due to its special design (e.g., the outer sealing skirt of the ES3) and presumed increased surface pressure and consecutive higher compression of the peripheral tissue[10]. As a limitation, patients with pre-implanted pacemakers were not excluded from the baseline cohorts, particularly those patients who were in the PARTNER trials[11][12]. A total of 22.9% (n = 35/153) of the patients in the PARTNER B trial and 11.7% (n = 118/1011) in the PARTNER 2 trial had pre-implanted pacemakers and were not excluded from the analysis regarding the new onset (PARTNER: 8/179 vs. 8/144; PARTNER 2: 85/1011 vs. 85/893) of pacemaker dependency following TAVR. This inclusion may lead to a relevant underestimation of pacemaker dependency following TAVR.

Most importantly, the characterization of frequent complications becomes increasingly important, especially in the case of further widening of the TAVR-indication to include intermediate risk patients. Younger and intermediate risk patients risk might experience more frequent pacemaker replacements due to their longer life expectancy, which would be associated with an increased risk of complications[13]. Some studies investigating possible predictors of pacemaker dependency have been published. However, interpreting these data is complex due to the vast parameter collections, different endpoints and small sample sizes in at least some of the studies. Other studies have merely investigated the occurrence of conduction disturbances such as new left bundle branch block (LBBB), whereas others have concentrated on the predictors of pacemaker dependency in one particular valve design[4].

There is confusion about the optimal management of PPI after TAVR, and hence, many centers started to implant PPI early after TAVR to avoid complications and to ensure postoperative mobilization and early discharge of the patients. This practice may be contradicted by the principles of individualized patient treatment and the “choosing wisely” paradigm. Therefore, the present work provides an insight and new evidence from an unselected, large, single-center, real-world cohort in which careful and individual decision-making was utilized regarding PPI following TAVR.

In this analysis of prospectively collected data related to TAVR and post interventional PPI, we also sought to elucidate the electrophysiological, procedural and clinical baseline parameters associated with PPI following TAVR in an all-comers real-world cohort with heterogeneous valve selections.

## 2. Methods

### 2.1 Patient population and study design

We analyzed the ECGs and clinical parameters of 494 consecutive patients treated with transfemoral (tf-) TAVR from April 2009 to August 2015 at our institution. Our study was approved by the local ethics committee (Ethic committee Göttingen: 22/4/11) and was conducted according to ICH-GCP standards–due to the retrospective character of the study, the need for consent was waived by our local ethic committee. Patients with in-hospital deaths, a previously implanted pacemaker, ICD or CRT systems and valve-in-valve procedures were excluded. Patients with already implanted devices were excluded because no differentiation between already existing conduction disturbances and those caused by TAVR would be performed. Patients who died in the peri-interventional phase were excluded for similar reasons. Patients with valve-in-valve procedures were excluded because pressure on the perivalvular tissue through TAVR is different in this situation. The assumed pathomechanism of conduction disturbances following TAVR differs in valve-in-valve procedures.

### 2.2 Procedures and decision making

Selection of the TAVR patients was performed by our local Heart Team consisting of interventional cardiologist, cardiac surgeons and anesthesiologists. All patients were implanted by one of three leading cardiologists together with a cardiac surgeon and treated using a standardized method according to the local protocol.

Different valve types and generations of valve types were implanted. Careful valve selection was completed individually for each patient by the Heart Team. Decisions for pacemaker implantations were performed assessing each indication, and the timing of the implantation was determined individually according to the current ESC guidelines[14]. Regarding point of time, there was no automatism or routinely pacemaker implant on same day. We considered an earlier PPI if patients had persistent conduction disturbances, predominant complete AV block, poor clinical stability due to e.g. delir, temporary pacemaker dislocation and medication. Accordingly, we considered later PPI if conduction disturbances were not continuously detectable, were asymptomatic, in some cases a “watch and wait” strategy was chosen for at least one day. If patients showed signs of an infection, PPI was postponed as well.

Patients were monitored on our intermediate care unit following TAVR procedure. Temporary pacemaker was removed at first post procedural morning if there were no signs of serious conduction disturbances in the continuous ECG monitoring. During the first 24 h following TAVR an invasive blood pressure monitoring, continuous ECG analyses and oxygen saturation were established. After 24 h patients were monitored with a continuous ECG solely until discharge.

### 2.3 Data collection

ECG examinations were routinely performed at admission and discharge. Clinical parameters as well as laboratory values were collected prospectively.

### 2.4 Statistical analysis

As part of the statistical analysis, the PR interval was dichotomized using a cut-off value of 178 ms. This cut-off value maximized Youden’s J index[15]. For calculating Youden’s J index, a PR interval higher than the cut-off was considered a predictor for requiring a pacemaker.

To identify variables that affect the probability of requiring a pacemaker after TAVR, a univariate logistic regression for each of the 42 identified risk factors was performed. The p-values and confidence intervals are reported. The variables with a p-value <5% in the univariate logistic regression were included in a multiple logistic regression. The missing values were then imputed using the multivariate imputation by chained equations (MICE) method[16](16). For the multiple logistic regression of the pooled results based on 300 imputations, the odds ratios, 95% confidence intervals, and two-sided p-values are reported. P-values smaller than 5% are referred to as statistically significant. Due to the explorative nature of these analyses the p-values were not adjusted for multiple testing. The time to pacemaker implantation is displayed by a Kaplan-Meier curve. All statistical analyses were performed in R version 3.3.3[17]. Graphics were done with Graph pad prism (LaJolla, CA, USA).

## 3 Results

### 3.1 Study population

**Fig 1:**
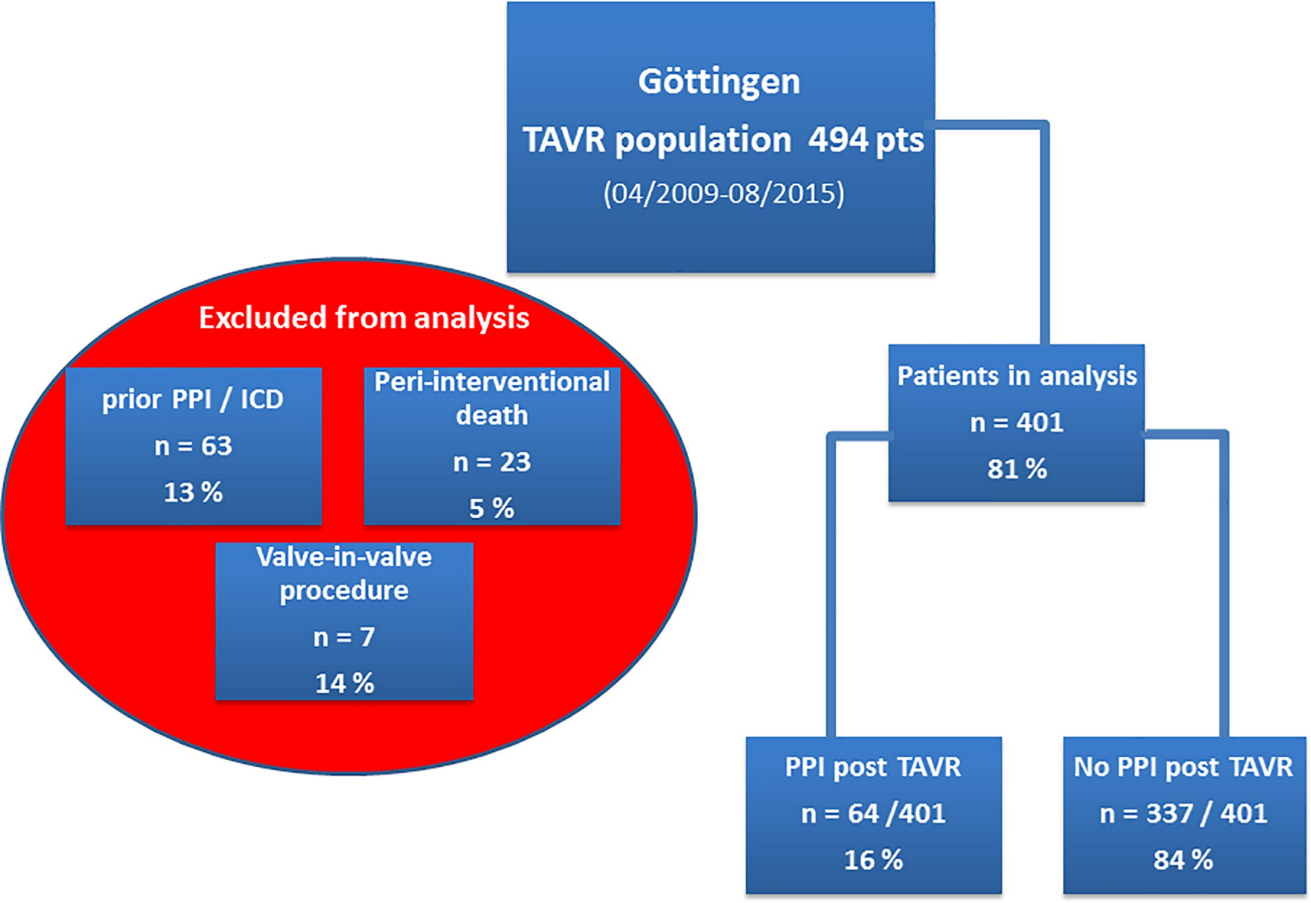
Analyzed cohort of 401 included patients and 93 patients excluded due to different reasons (prior pacemaker or ICD implantation, peri-interventional death and valve-in-valve procedures).

Over a period of six years, a total of 494 patients were treated with tf-TAVR at our institution. A total of 93 patients were excluded from the analysis for the following reasons: a previously implanted pacemaker was present in 63 cases (13%), peri-interventional death occurred in 23 cases (5%) and a valve-in-valve procedure was performed in 7 cases (14%) (Fig 1). Therefore, 401 patients were analyzed with regards to pre- and post-procedural ECG parameters, risk scores and baseline parameters. Only pre-procedural ECG parameters were used for the analyses of the PR interval.

The cohort included “typical” TAVR patients characterized by high age (mean age 82 years) and high perioperative risk (Log. EuroScore 20.5; EuroScore II 5.8, STS score 5.5) (Table 1).

**Table 1:** Characteristics of the patients at baseline (yr = years; CABG = coronary artery bypass graft; COPD = chronic obstructive pulmonary disease; GFR = glomerular filtration rate).

Patient characteristics at baseline	
Age—yr	82 ± 5.1
Male sex	139 (34.7)
Log. EuroScore I	20.5 ± 13
Log. EuroScore II	5.8 ± 5
Body-mass index	26.9 ± 5
STS risk score	5.5 ± 3
Coronary artery disease	265 (66.1)
Previous CABG	41 (10.2)
Previous PCI	121 (30.2)
Cerebral ischemic event	56 (14.0)
Peripheral vascular disease	67 (16.7)
Diabetes mellitus	132 (32.9)
COPD	72 (18.0)
Atrial fibrillation	192 (47.9)
paroxysmal	81 (20.2)
persistent	26 (6.5)
permanent	85 (21.2)
Renal failure	211 (52.6)
GFR < 30	40 (10.0)

Mean ± standard deviation or frequency (%)

A PPI was required in 64 out of 401 patients (15.9%). The main indications were complete AV-block, AV-block IIb (Mobitz) and symptomatic bradycardia with a mean duration time to pacemaker implantation of 5.4 ± 4 days (median 5 days). Approximately two-thirds of the treated population suffered from coronary artery disease (CAD), and nearly 40% had a past medical history of coronary artery bypass graft (CABG) or percutaneous coronary intervention (PCI) prior to the TAVR procedure. A history of atrial fibrillation was present in 192 patients (47.9%). Impaired renal function was diagnosed in 211 patients (52.6%) (Table 1).

### 3.2 Valve selection and pacemaker rates

**Fig 2:**
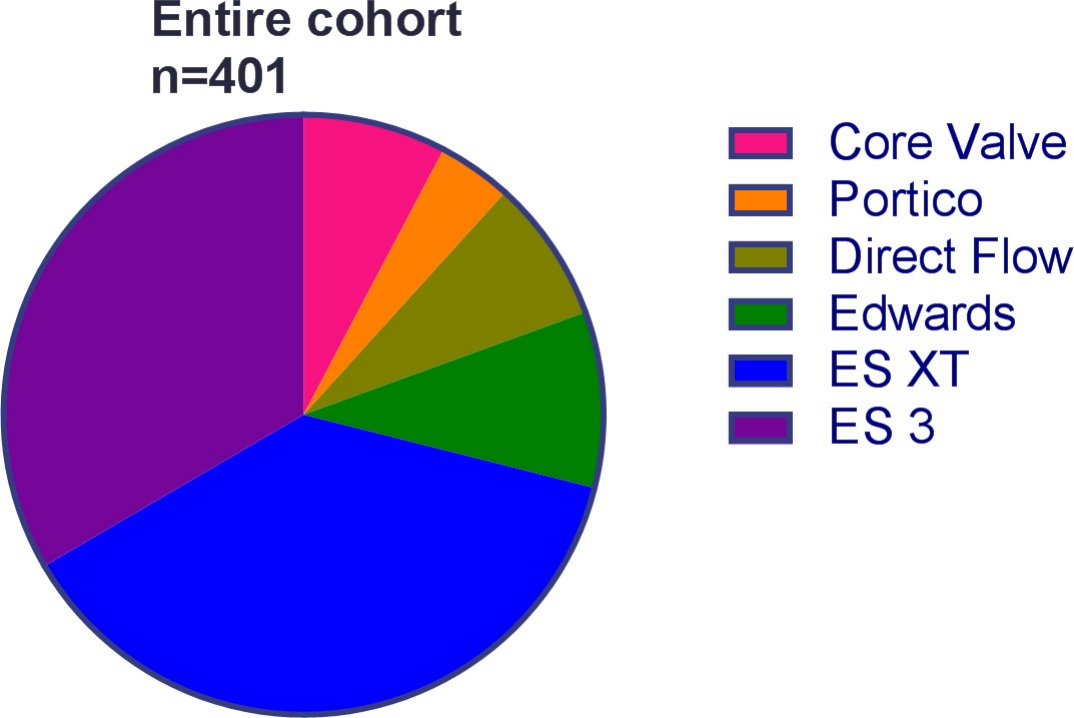
Distribution of the different valve types in the entire cohort (n = 401).

The following valve types were implanted: Edwards SAPIEN (Edwards Lifescience, Irvine, CA, USA) in n = 38 (9.4%), Edwards SAPIEN XT (Edwards Lifescience, Irvine, CA, USA) in n = 151 (37.6%), Edwards SAPIEN 3 (Edwards Lifescience, Irvine, CA, USA) in n = 134 (33.4%), CoreValve System 1^st^ Generation (Medtronic, Minneapolis, MN, USA) in n = 31 (7.7%), Portico (St. Jude Medical, Saint Paul, MN, USA) in n = 16 (4.0%) and Direct Flow (Direct Flow Medical, Santa Rosa, CA, USA) in n = 31 (7.7%) (Fig 2).

**Fig 3:**
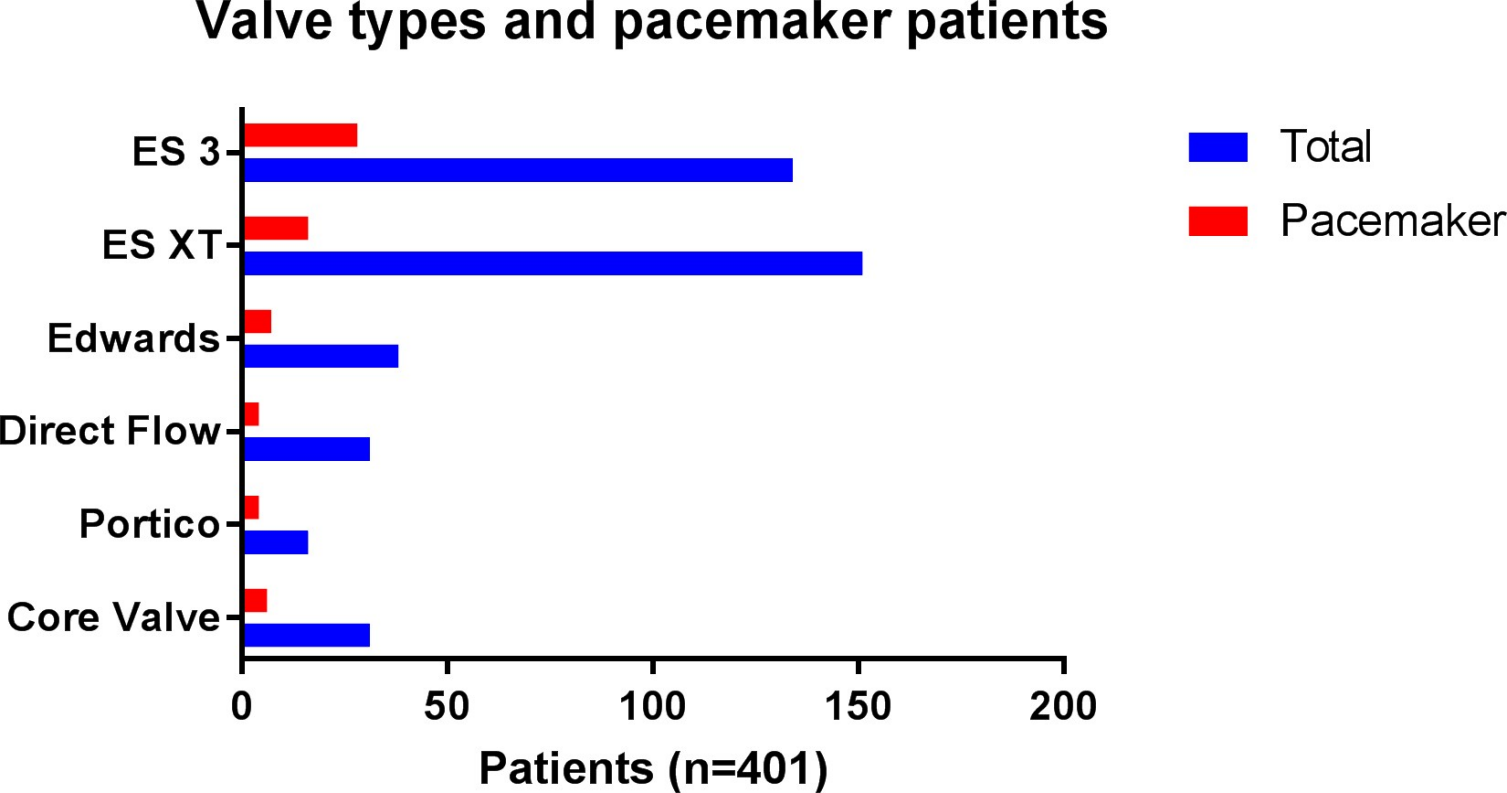
Different valve types with total implantation number and proportion of pacemaker patients (n = 401).

There was no statistically significant difference regarding PPI rates between valve types [Edwards SAPIEN 18.4% (7 PPI in 38 TAVR procedures) vs. Edwards SAPIEN XT 10.5% (16/151) vs. Edwards SAPIEN 3 20.8% (28/134) vs. CoreValve System 1^st^ Generation 19.3% (6/31) vs. Portico 25% (4/16) vs. Direct Flow 12.9% (4/31)] or valve sizes in our analysis (Table 2; Fig 3).

**Table 2:** Results of univariate analysis of baseline clinical, ECG and procedural parameters.

Clinical baseline parameters	Mean	Standard deviation	p—value
- Age (years)	82.2	5.2	0.724
- EuroScore I	19.2	12.6	0.067
- EuroScore II	5.5	5.1	0.578
- STS score	5.4	3.2	0.512
- BSA	1.8	0.2	0.772
- Valve type	NA	NA	0.159
- Valve size	NA	NA	0.318
- LVEF (cut off 50%)	NA	NA	0.508
- Coronary artery disease	NA	NA	0.091
- Prior PCI	NA	NA	0.467
- CABG	NA	NA	0.696
- Peripheral vascular disease	NA	NA	0.969
- Cerebral ischemic disease	NA	NA	0.790
- COPD	NA	NA	0.762
- Diabetes	NA	NA	0.433
- Chronic kidney disease	NA	NA	0.504
- GFR < 30 ml/min	NA	NA	0.392
- GFR < 60 ml/min	NA	NA	0.085
- Creatinine (mg/dl)	1.18	0.6	0.328
**Laboratory values**			
- Hemoglobin (mg/dl)	12.3	5.8	0.558
- Sodium (mmol/l)	139.2	3.8	0.206
- Potassium (mmol/l)	4.0	0.5	0.614
- TSH (mU/l)	1.1	1.1	0.521
- Creatine kinase (U/l)	94.7	139.8	0.638
- Difference in Creatine kinase (U/l)	30.0	174.6	0.790
**Procedural parameters**			
- Length of procedure (min)	68.6	34.9	0.217
- Amount of contrast medium (ml)	140.9	58.9	0.444
- Fluoroscopy time (min)	15.1	9.6	0.867
- Post dilatation	NA	NA	**0.002**
**ECG parameters**			
- Resting heart rate (bpm)	74.1	14.6	**0.026**
- QRS time (continuous)	101.9	21.9	**0.020**
- AV-block I°	NA	NA	0.486
- PR interval (cut-off> 178 ms)	NA	NA	**0.003**
- Left anterior fascicular block	NA	NA	**0.001**
- Left bundle branch block	NA	NA	0.194
- Right bundle branch block	NA	NA	**0.001**
- Pathological electrical heart axis	NA	NA	**0.034**
- Change in electrical heart axis	NA	NA	0.723
- Atrial fibrillation	NA	NA	0.077

### 3.3 Comparison of clinical parameters of patients with and without the need for a pacemaker following TAVR

Eligible patients were divided into two groups: patients with an indication for a pacemaker vs. patients without an indication for a pacemaker following TAVR.

Multiple clinical parameters (patients’ medical history, laboratory blood tests, peri-interventional measurements) were analyzed with regards to differences between the patients requiring pacemaker therapy and those who did not (Table 2). Post dilatation (intraprocedural) to attenuate paravalvular leakage was significantly more common in patients requiring PPI (50% (32/64) vs. 27.8% (94/337); p = 0.002). A history of atrial fibrillation (p = 0.077) and mild to moderate renal impairment (GFR 30–60 ml/min; p = 0.085) showed a trend but did not reach statistical significance. Other clinical parameters such as age, different risk score models (EuroScore I+II, STS score), amount of contrast medium, or the total procedure time were not of statistical significance (Table 2).

### 3.4 Comparison of ECG parameters of patients with and without the need for a pacemaker following TAVR

Lower resting heart rate (as a continuous variable; p = 0.026), QRS duration (as a continuous variable; p = 0.02), a PR interval cut off value of >178 ms (p = 0.003), left anterior fascicular block (p = 0.001), a pre-existing RBBB and pathological electric heart axis (marked left and right axis deviation and right axis deviation; p = 0.034) significantly affect the probability of a pacemaker implantation.

### 3.5 Multiple logistic regression analysis

Statistically significant parameters in the univariate analysis as well as the medically justified interactions between LAFB and QRS, between LBBB and QRS, and between RBBB and QRS were tested in a multiple regression model. The interaction between LAFB and RBBB/LBBB could not be estimated. None of the listed interactions was significant. Therefore, we present the model without interactions (Table 3)). In this model, post dilatation (OR 2.219; 95% CI (1.106; 3.667); p = 0.007) and a PR interval above 178 ms (OR 0.412; 95% CI (1.058;5.134); p = 0.027) remained as independent predictors for PPI therapy following TAVR. Other parameters were no longer significant in the multiple regression analysis (resting heart rate p = 0.126; QRS time p = 0.473; left anterior fascicular block p = 0.063; left and RBBB, p = 0.190 and p = 0.208, respectively).

**Table 3:** Multiple logistic regression analysis of predictors of permanent pacemaker dependency (n = 401).

	Odds ratios (OR)	Lower and upper confidence interval for OR	p-value
(Intercept)	0.177	0.013; 2.427	0.195
QRS duration (continuous variable)	1.007	0.988; 1.027	0.464
Left anterior fascicular block	4.966	0.779; 31.658	0.090
Left bundle branch block	0.301	0.045; 2.024	0.217
Right bundle branch block	2.174	0.687; 6.885	0.187
Post TAVR dilatation	2.014	1.106; 3.667	**0.022**
Resting heart rate (continuous variable)	0.984	0.963; 1.005	0.144
PR interval (cut off 178 ms)	2.331	1.058; 5.134	**0.0358**

### 3.6 Post procedural time to pacemaker implantation

**Fig 4:**
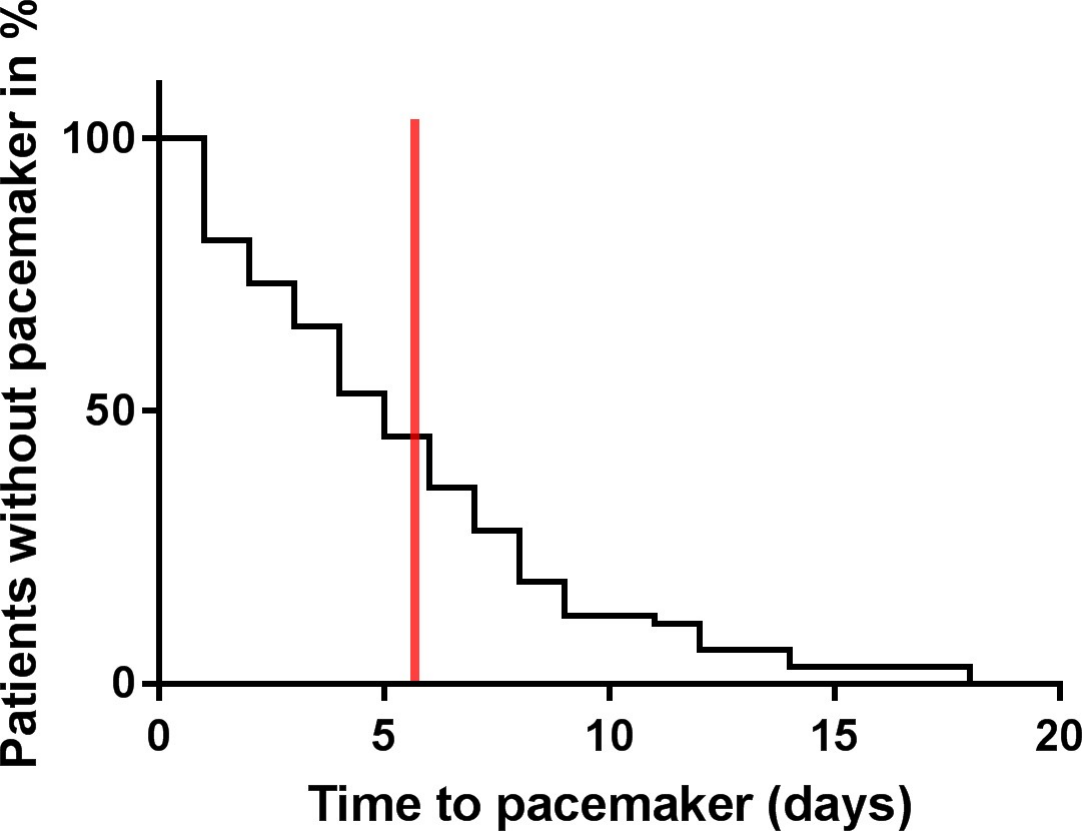
Kaplan-Meier analysis of PPI following TAVR in the pacemaker group (n = 401). Mean length of index procedure hospital stay (5.75 days) in the SURTAVI trial[6] marked with a red line.

The mean time to pacemaker implantation following TAVR was 5.5 days. Fig 4 shows the earliest implantation on day 1 post intervention and the latest implantation on day 18 post TAVR. For comparison, the mean length of the index procedure hospital stay of the most recent TAVR trial was 5.75 days and is marked with a red line(6).

## 4 Discussion

### 4.1 Pacemaker implantation after TAVR

We sought to elucidate the electrophysiological, procedural, and clinical baseline parameters that are associated with and perhaps predict the need for permanent pacemaker implantation after TAVR in a heterogeneous-valve-type real-world cohort. The key feature of our study is that, unlike other published studies, we examined a large sample size of 401 consecutive patients with a variety of valve types, and our data therefore more closely represent a typical real-world cohort.

Our patients are comparable to other published TAVR cohorts [3] characterized by high age and being a high risk population (82 vs. 81 years; EuroScore I 19.2 vs. 18.3 STS 5.4 vs. 5), and our cohort represents a typical collective treated in accordance with the current guidelines[1].

It may be assumed that the risk of PPI following TAVR differs between each valve type; therefore, specific trials would be ideal to individualize TAVR therapy. Nevertheless, TAVR is a rapidly disseminating therapy performed in a growing number of hospitals in different countries with different social and health economic infrastructure. Therefore, regional availability of valve types may vary, which may support the utility of our study.

A PPI rate of 15.9% is comparable to other published cohorts consisting of various valve types of different generations and manufacturers[3][4]. The time to PPI implantation (5.5 days) in our study is also comparable to large TAVR cohorts as well as the PARTNER trial and registry, which had a mean time to implantation of 4.1 days[5]. This result may reflect the careful and individualized decision making undertaken for each patient. Nevertheless, the PPI rates differ between real-world collectives, such as our cohort, and others, such as selected collectives like the PARTNER trials, in which the PPI rate was markedly lower (8.5% in PARTNER 2)[12][18][19]. The difference between implantation rates in general is not completely clear. Future studies should prospectively examine the role of a specific valve in this context.

A meta-analysis by Siontis et al. reported the predictors of PPI following TAVR in 11,210 patients from 41 trials (average of 273 pts. per study) [4]. Of note, our study would have been the largest single-center cohort in this analysis. They found first-degree AV-block, left anterior hemiblock, RBBB and male sex to be significant predictors of PPI. Most of the risk factors identified in this meta-analysis are mainly confirmed by our results from this real-world cohort. The first-degree AV-block was not significant in our population; however, we did show that a longer PR interval is associated with an increased risk of PPI, indicating that AV-block may be a significant factor in a larger sample size. The same might be true in patients with a pre-existing left anterior hemi block, which reached statistical significance in univariate analysis. The multivariate analysis resulted in a high odds ratio, but statistical significance was not reached. The presence of RBBB was also a significant predictor in the univariate analysis of our cohort.

### 4.2 Post TAVR balloon dilatation

Post TAVR balloon dilatation is required if the valve was not expanded sufficiently and/or a relevant regurgitation was still detectable. In this case, the operator further expands the TAVR device with a balloon to minimize aortic regurgitation. Pre- and post-balloon dilatation were tested as predictors for the need for PPI following TAVR and pre-balloon dilatation analyses remain contradicting. Post- balloon dilatation failed to show significant impact on pacing following TAVR in an isolated Lotus valve cohort with 228 patients, and a remarkable PPI rate of 32% with a median time to PPI of 3.0 days. [20][21][22]. It was presumed that pre-dilatation may avoid the need for post-dilatation(17). However, since there was a change in our local protocol regarding the performing of pre-dilatations, we could not prove this assumption with our data. In our analysis, balloon dilatation after TAVR proved to be a significant predictor of PPI. However, significant paravalvular regurgitation (PVR) is a well-accepted risk factor for a poor outcome following TAVR[7], and interventionalists may rather accept an increased risk for PPI post TAVR. Nevertheless, our result indicates and strengthens the already described association between mechanical effects of TAVR with consecutive tissue trauma and conduction disturbances[21]. However, on the basis of different published research in this controversial field, it is not clear if there exists a difference in prognosis between the grades of PVR. Some authors even suggested that mild PVR could be accepted in selected patients[7][23][24]. Therefore, depending on future data, post TAVR balloon dilatation may be carefully evaluated in patients with only mild PVR due to the higher risk of PPI in these patients.

### 4.3 ECG parameters

The aortic annulus is located near the AV node and the cardiac conduction system, and the supraventricular structures may be especially influenced by the implantation pressure. In light of this fact, there is evidence that pre-existing conduction abnormalities such as AV-block I, RBBB or LBBB as well as left anterior fascicular branch block may predict conduction disturbances following TAVR[10]. We also detected a significant effect of a PR interval of above 178 ms on the need for PPI following TAVR. We are not aware of any literature describing a cut-off value in PR interval, although a PR interval with a cut-off value of 200 ms (first degree AV-block) was found to be a predictor of PPI[18]. In our statistical analysis, the PR interval was dichotomized using a threshold of 178 ms. This threshold maximized the Youden index, in other words, it maximized the sum of sensitivity and specificity as a predictor of post-TAVR PM implantation. Other statistical methods for determining the optimal threshold could have been considered, for instance the Euclidean distance, the product of sensitivity and specificity, the accuracy, or the diagnostic odds ratio[25]. Siontis et al. reported in their meta-analysis of 11,210 TAVR patients with a heterogeneous valve selection that first degree AV-block was a significant predictor of PPI after TAVR[4]. Other studies analyzed only the Edwards SAPIEN or the CoreValve and did not find first degree AV-block to be a significant predictor of PPI, but they did not analyze the PR interval as a continuous variable[5][21]. Accordingly, first-degree AV-block was not significant in our analysis either, although it may be assumed that a prolonged PR interval is associated with PPI therapy following TAVR.

Other ECG parameters such as RBBB or left anterior fascicular branch block proved to be significant in other analyses[4][5]. Although these factors were statistically significant in the univariate analysis, the results failed to remain significant when inserted in the multiple regression model. This finding may be mainly driven by the strong influence of the post TAVR balloon dilatation in our analysis. Post balloon dilatation and its potential influence on ECG parameters were not demonstrated in the other analyses mentioned. These discrepancies may also be explained by differences in sample size with our sample being substantially larger than previous investigations.

### 4.4 Other parameters

No laboratory parameters known to have a potential impact on the cardiac conduction system, e.g., potassium, hemoglobin or thyroid stimulating hormone, showed any correlation with PPI, which indicates a pure mechanical pathomechanism as the cause of conduction disturbances following TAVR.

Furthermore, we did not detect any association between any of the present comorbidities or age with the typical surgical risk scores (EuroScore, STS-Score) and PPI following TAVR. This fact also supports the theory of a pure mechanical pathomechanism and a limited regional problem of the peripheral structures of the aortic valve.

### 4.5 Widening of TAVR indication

TAVR is performed around the world with similar results in terms of safety and efficacy. Thus, there has been a recent increase is interest in expanding the TAVR indication to include intermediate and low risk patients[12]. In this context, PPI as a typical complication gains particular importance. Younger and low-risk patients are typical surgical candidates, and the guidelines recommend surgical aortic valve replacement in these patients. Pacemaker rates in surgical candidates are lower than in published TAVR cohorts (4% GARY surgery vs. GARY interventional 17.5%)[3][26] In younger patients in particular, a likely change of pacemaker during the lifetime must be considered, and these younger patients have to be educated regarding this additional risk during the decision making process[13][27][28][29]. In this context, it is of particular importance to investigate this issue, and it is especially important to determine the predictors of PPI after TAVR.

### 4.6 Impact on daily practice

Our results reflect one of the largest single-center cohorts and combine baseline, ECG, and procedural parameters with regard to their influence on PPI post TAVR. Patients with a baseline PR interval of > 178 ms might be informed (prior to the TAVR procedure) about their higher risk of developing serious conduction disturbances, possibly leading to a PPI after TAVR. Patients who need intraprocedural post dilatation are at increased risk for developing serious conduction disturbances in the post procedural period. These particular patients may need to be evaluated more carefully because PPI is more likely. Furthermore, patients with the above described baseline, ECG, and procedural characteristics who do not develop conduction disturbances in the very early post interventional phase should be observed in-hospital for up to 7 days post implantation according to the current guidelines due to their high risk for developing conduction disturbances[14]. As shown in Fig 4, there are patients with late and very late development of conduction disturbances. This work does not provide any solution for this problem but does show that hospital discharge 5 days after the procedure may be too early to detect conduction disturbances in some patients. Furthermore, identifying patients who are at a high risk of PPI appears to be helpful in avoiding prolonged hospitalization.

### 4.7 Limitations

The present paper contains data of a single center with prospectively collected data. Therefore, our conclusion is not more than hypothesis generating. Representing a real-world cohort of a high performing single center in Europe the valve selection was not balanced and reflects a kind of learning curve in using different manufacturers of transfemoral aortic valves.

## 5 Conclusion

We investigated predictors of PPI in a large single-center cohort with a heterogeneous valve selection. Post balloon dilatation of the TAVR prosthesis as a novel predictor and a prolonged PR interval were associated with a significant higher risk of PPI. This should be taken into account when post dilatation is considered.

## Supporting information

S1 Table Supporting information file–database excel sheet “Insights into permanent pacemaker implantation following TAVR”. (XLSX)
